# Monitoring Inattention in Construction Workers Caused by Physical Fatigue Using Electrocardiograph (ECG) and Galvanic Skin Response (GSR) Sensors

**DOI:** 10.3390/s23177405

**Published:** 2023-08-25

**Authors:** Yewei Ouyang, Ming Liu, Cheng Cheng, Yuchen Yang, Shiyi He, Lan Zheng

**Affiliations:** 1 Key Laboratory of Physical Fitness and Exercise Rehabilitation of Hunan Province, College of Physical Education, Hunan Normal University, Changsha 410012, China; 2 Department of Architecture and Civil Engineering, City University of Hong Kong, Kowloon, Hong Kong, China

**Keywords:** HRV, ECG, GSR, inattention, physical fatigue

## Abstract

Physical fatigue is frequent for heavy manual laborers like construction workers, but it causes distraction and may lead to safety incidents. The purpose of this study is to develop predictive models for monitoring construction workers’ inattention caused by physical fatigue utilizing electrocardiograph (ECG) and galvanic skin response (GSR) sensors. Thirty participants were invited to complete an attention-demanding task under non-fatigued and physically fatigued conditions. Supervised learning algorithms were utilized to develop models predicting their attentional states, with heart rate variability (HRV) features derived from ECG signals and skin electric activity features derived from GSR signals as data inputs. The results demonstrate that using HRV features alone could obtain a prediction accuracy of 88.33%, and using GSR features alone could achieve an accuracy of 76.67%, both through the KNN algorithm. The accuracy increased to 96.67% through the SVM algorithm when combining HRV and GSR features. The findings indicate that ECG sensors used alone or in combination with GSR sensors can be applied to monitor construction workers’ inattention on job sites. The findings would provide an approach for detecting distracted workers at job sites. Additionally, it might reveal the relationships between workers’ physiological features and attention.

## 1. Introduction

Staying focused is quite important for construction workers to ensure safety in hazardous and constantly changing construction workplaces. Distraction is a major cause of the occurrence of construction workers’ unsafe behaviors [1], e.g., inattention to certain hazard types has been identified as one of the most common reasons that lead to failed hazards identification [2]. Meanwhile, construction workers may frequently suffer from physical fatigue since the construction industry involves lots of physically demanding tasks [3]. Physical fatigue is defined as a reduced capacity to perform physical work resulting from activities requiring physical effort [4]. It may cause task distraction, which would increase human errors at workplaces, and, thus, the number safety incidents that occur [5,6]; e.g., an interview investigation reported that negligence and distraction were two leading causes of falling from height among construction workers [7]. Therefore, it is crucial to maintain focus on workers’ attentional state in relation to physical fatigue to ensure their safety.

The physiological data, which include heart rate variability, eye movement, electromyography, galvanic skin reaction, and brain activity, are a better way to show the subjects’ physiologically dependent cognitive states. It is an ideal approach for cognition measurement, providing objective data, having little or no interference with task execution, and providing information continuously without significant delay [8]. Among these physiological measurements, neural signals provide a direct measure of brain status transformation, which is suggested to be the most suitable human physiological signal processor for evaluating human attention. Particularly, electroencephalography (EEG) is a neuroimaging technique that directly measures the brain’s state with a relatively high temporal resolution. EEG has been widely applied to indicate attention [9]. However, there are severe limitations if using EEG for real-time monitoring in construction workplaces, e.g., subjects must remain relatively stable and quiet, and necessary equipment is invasive and complex. In addition, EEG signals are easily hindered by the surrounding noise.

Peripheral physiological signals like an electrocardiograph (ECG) and the galvanic skin response (GSR) are more accessible to collect than neural signals in construction workplaces and thus may be more feasible. Moreover, they can indicate human response to physiological arousal; thus, ECG and GSR signals would be potential biomarkers for practical inattention monitoring [10,11]. More importantly, these signals are easier to collect and analyze than EEG. The fast-developing unobtrusive sensors, such as wristbands, armbands, chest straps, and embedded sensors [12], enhance their feasibility in real-life conditions. Therefore, in order to investigate the prediction abilities of the ECG and GSR signals in monitoring workers’ inattention due to physical fatigue, this study aims to develop prediction models using supervised learning algorithms with ECG and GSR signals as input features. The findings would provide an approach for detecting distracted workers at job sites. Furthermore, they could also reveal the relationships between workers’ physiological features and attention.

## 2. Literature Review

### 2.1. Physical Fatigue and Inattention

Physical fatigue might cause spontaneous distraction in humans [13]. The human body’s increased muscular activity and cardiovascular responses due to physical activity would trigger physiological arousal [14]. It is well established that arousal influences cognitive performance, following an inverted U-shaped relationship [15]. That is, moderate arousal levels facilitate performance, while low or high arousal levels would impair performance. The study conducted in [15] also reported that light physical activities may improve cognitive function, whereas heavy physical activities may impair cognitive performance, like distraction. Previous research on specific groups of people who engaged in work requiring good attention (e.g., pilots and drivers) found that these staff reported difficulty concentrating and remaining attentive during tasks when fatigued [16].

In addition, physical activity may also reduce human inhibitory control due to competition for shared processes, e.g., the central executive process between concurrent physical and cognitive tasks [17,18]. Park et al. [19] found that fatigued subjects were more vulnerable to distractor interference during visual search tasks, potentially due to reduced inhibitory control. It has also been demonstrated [20] that fatigued subjects avoid attention that demands controlled processing.

### 2.2. Attention and Occupational Safety

Attention allows us to bias the processing of incoming information so that we can focus on the information relevant to achieving the current goals and actively ignore irrelevant information that might interfere with those goals [21]. Inattention is also known as mind-wandering and distraction. The common thread of inattention is that people divert their attention from the primary task to something else instead of focusing on the task at hand. This is because when a human’s limited amount of attentional resource is diverted to a distractor, performance in the primary task that requires attention often deteriorates [22].

The outcomes of inattention are severe in construction, which is the primary contributor to work errors and possible injuries. Construction workplaces involve many safety hazards, and unrecognized hazards have been labeled as “root causes” [2]. If workers recognize safety hazards, they are more likely to reduce injury likelihood. Concentration is essential for hazard identification, and the degree of attention and the spread of visual attention during hazard search operations can affect hazard recognition levels [23]. In contrast, selective attention (i.e., filtering and selecting useful information for further processing) or inattention to certain hazard types has been identified as one of the most common reasons that lead to unrecognized hazards [2,24]. Hinze’s distraction theory also [25] suggests that workers distracted by productivity demands are more likely to overlook safety hazards. The study performed in [26] also demonstrated that distracted workers recognized a smaller proportion of hazards than undistracted workers.

### 2.3. Inattention Measurement

Methods for measuring inattention include subjective, behavioral, and physiological measures. Subjective measures usually refer to self-assessment scales or interviews. There have been typical scales for measuring human cognitions, e.g., the mindful attention awareness scale [27] and the mind wandering scale [28]. However, relying on subjects’ self-reports is limited because a person’s actual objective state is different from the subjective feeling of their state; also, collecting a worker’s self-assessment is burdensome and impractical on construction sites.

Subjects’ behavioral responses in specific tasks can also indicate concentration. For example, speed variability and lane deviation can serve as behavioral detections to seize driving distractions [29]. Nevertheless, no universal performance metrics proposed for attention levels can apply to construction programs as construction tasks and scenarios differ from those of driving.

Physiological measures have plenty of advantages. Physiological parameters have better accuracy and reliability since physiological responses cannot be controlled arbitrarily [8]. Physiological measures can also function continuously. It is promising to monitor construction workers’ attention in workplaces with minimal interference to construction activities. Among human physiological signals, EEG directly measuring the brain’s state has been widely applied to indicate distraction (e.g., [30]). However, it is not easily applied on job sites. The EEG device is also challenging to operate, and subjects must maintain reasonable stability and silence. Additionally, the noise in construction workplaces can easily interfere with EEG signals.

Peripheral physiological signals might address the limitations of EEG sensors. ECG indicates the heart’s rhythm and electrical activity by attaching sensors to the skin and detecting the electrical signals produced by the heart each time it beats. ECG signals can be collected through wristbands, armbands, or chest straps. It has been found that there is a strong correlation between ECG signals and attention [31]. The study [32] also reported that ECG signals exhibited distinct patterns as the subjects performing tasks demanded different attention. Moreover, heart rate variability (HRV) derived from ECG signals can indicate the patterns of the autonomic nervous system (ANS) [33]. ANS is divided into sympathetic and parasympathetic nervous systems. The sympathetic nervous system is active when we meet stress, fear, and emergency status, while the parasympathetic system will be active when we are relaxed. Hence, HRV can reflect various physiological states, including sustained attention to tasks. Whether a person is in sustained attention or in a relaxed situation will reflect in the heart rate variability change [34]. More specifically, the study [35] reported that the power of a very low-frequency band and the mean of RR were the best indicators. The study in [36] reported that the 0.10 Hz component of HRV frequency feature may be considered a psychophysiological index of effort allocation. The results of [37,38,39] also indicated that entropy ECG signals were sensitive to driving distraction.

GSR is a nonobtrusive modality and easy to collect. It has already been included in consumer electronics such as wristwatches. Electrodermal activity originates from the activation of the sweat glands in response to physiological arousal or stress [10]. It can also infer changes from the sympathetic nervous system and thus is widely used in physiological measurements [40]. For example, skin conductance will rise when a psychological state causes a human to experience stress or arousal. GSR has a long history of use as an indicator of attention [41]. It has been shown to have a meaningful relationship to visual attention [21] and can be used to detect distraction under naturalistic driving conditions [42,43,44]. GSR sensors have also been widely used for intervention in people with autism spectrum disorder [45]. There were also studies reporting reduced skin conductance levels [46] and a reduced number of non-specific skin conductance responses [47] when subjects are inattentive.

## 3. Methodology

### 3.1. Experimental Design

An experiment was designed to achieve the research objectives. Subjects completed tasks requiring concentration in physically fatigued and non-fatigued states, capturing their physiological (ECG and GSR signals) and behavioral data. Supervised machine-learning methods were applied to develop prediction models indicating workers’ inattention, where features extracted from ECG and GSR signals served as training data. The detailed experimental design is clarified below.

#### 3.1.1. Subjects

A total of 30 males volunteered for the study. The participants were screened for visual health. They were construction workers aged 21 to 42 (M = 29.4 years; SD = 6.2 years). The participants were asked to maintain their sleeping habits, avoid vigorous physical activity, and avoid caffeine and nicotine intake for the 24 h preceding the experiment. The Ethical Research Committee at the City University of Hong Kong approved the study. Moreover, informed written consent was obtained from each participant.

#### 3.1.2. Apparatus

ECG was measured with the Custo Guard (Custo Med GmbH, Ottobrunn, Germany) in a quiet room. One electrode of the holter was placed in the fourth intercostal space just to the right of the sternum. GSR signals were collected using sensors from Shimmer (Shimmer Research, Dublin 11, Ireland). The sensors are wearable, and data are transmitted to the computer via Bluetooth. The signals are collected using ConsensysPRO (v 1.6.0). The GSR suit includes a sensor, two electrodes worn on the subjects’ left hand’s index finger and middle finger, and a strap for fixing the device on one’s wrist, with a frequency rate of 10 Hz.

#### 3.1.3. Experiment Task

The participants’ physical fatigue was induced by a manual material handling task. They carried a 15 kg sandbag, with continuous walking (4 km/h) on a treadmill. The specific mass of 15 kg was the average mass of material being handled by the workers on a construction site [48]. This material handling task is a good simulation of the actual construction work, which has been applied by many previous research studies [49,50,51,52,53]. Borg’s rating of perceived exertion (RPE) was adopted for measuring the participants’ physical fatigue levels. Borg’s RPE is a scale for quantifying subjective feedback on how tired subjects feel while performing physically demanding tasks [54]. It has been widely used as a valid tool to enable people to rate the level of fatigue they experience [55]. Specifically, Borg’s RPE is a linear scale, from 6 to 20, with descriptions ranging from “Very, very light” to “Very, very hard”, respectively [54]. Participants reported their fatigue level every two minutes. Participants stopped physical tasks when their RPE reached 17 (corresponding to high fatigue) because high fatigue would disrupt concentration [25].

**Figure 1:**
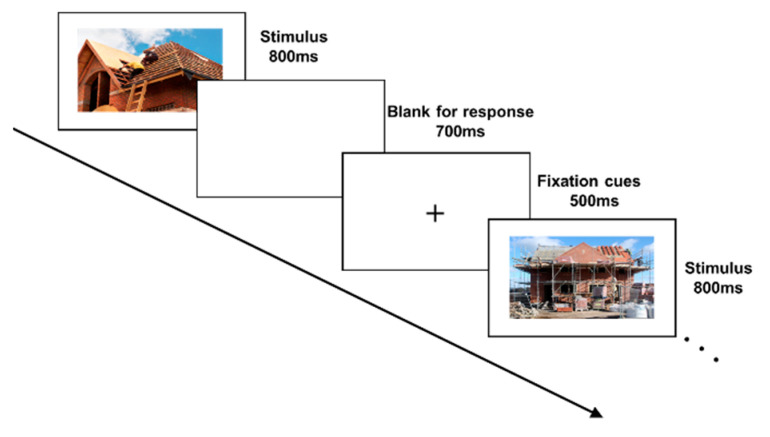
Design of the cognitive task.

Participants also performed a cognitive task inducing their attention. The cognitive task is a hazard identification task following the continuous performance test (CPT) paradigm. CPT is a psychological task widely used for attention/vigilance measurement [56]. This is also similar to workers being aware of workplace hazards. Specifically, participants identified construction hazards within the stimulus on the computer screen. The task included 150 stimuli, 20% containing hazards (targets), including common hazards in job sites like falls, struck-by, electric shock, and fire hazards. As shown in Figure 1, the stimulus duration was 800 ms, following a random presentation order. A blank view lasting 700 ms after the stimulus was included for participants’ response. The space key should be pressed if the current stimulus is a target. The subsequent stimuli appeared after 500 ms fixation cues. The whole task lasted 5 min, with E-Prime 3.0 running the experiment and recording participants’ keyboard responses [57].

#### 3.1.4. Experiment Procedure

Each subject performed the cognitive task twice on two separate days. One was performed under non-fatigued states, and another after subjects became physically fatigued. To avoid sequential effects, the 30 subjects were randomly divided into two equal groups and completed the tasks with different sequences, with one group performing the non-fatigued condition first and the other performing the fatigued condition first. Under the non-fatigued condition, subjects performed the cognitive task in a relaxed state. Regarding the fatigued condition, they completed the physical task at first and then performed the cognitive task.

Participants first received the briefing of the experiment, and they were given unlimited time to practice the cognitive task until they fully understood the experimental tasks. During the experiment, participants wore both ECG and GSR sensors to collect their physiological data simultaneously, sitting comfortably in front of a 15-in. laptop with a 1920 × 1080 pixels screen resolution. The experimenter monitored data recording during the whole process to ensure data quality. In the end, participants assessed their attention level through a five-point Likert scale ranging from 1 (constantly distracted) to 5 (very concentrated).

### 3.2. Physiological Signal Preprocessing and Feature Computation

ECG signals were imported into Kubios Premium (v3.5.0) [58] for noise removal, and HRV featured computation. The Pan–Tompkins algorithm [59] was applied to detect R-wave time instants. Additionally, bandpass filtering was used to reduce power line noise, baseline wander, and other noise components. The power spectral density was calculated by the Lomb–Scargle periodogram [60]. Based on original research articles [31,35,36,37,42,47,61,62,63], 14 HRV features listed in Table 1 that might be the potential to differentiate concentration and distraction were computed.

RR interval refers to the time gap between two consecutive normal R peaks of the ECG signal. These time domain features could indicate changes in the sympathetic nervous system (SNS) and parasympathetic nervous system (PNS); e.g., RMSSD estimates the vagally mediated changes reflected in HRV [64]. In addition, three main spectral components (VLF, LF, and HF) and TP were analyzed. VLF and HF could reflect PNS activity, LF relates to both the PNS and SNS, and TP measures the overall autonomic activity [64]. LF and HF were also measured in normalized units (n.u.). The ratio between LF and HF indicating the balance between parasympathetic and sympathetic activity [65] was also used. Regarding non-linear features, SD1/SD2 reflecting sympathetic activation [64] and two entropy (approximate entropy and sample entropy) measuring irregularity or complexity of RR series were included.

The raw GSR signals were imported to Ledalab (a Matlab toolbox) for preprocessing and feature computation. GSR includes a tonic and a phasic component. The tonic component changes slowly over time and is unaffected by sudden stimuli. The phasic component manifests as abrupt and transient increases in the skin conductance signal, resulting from external stimuli typically associated with stress or arousal. Commonly, the tonic component is referred to as the skin conductance level (SCL), whereas the short-term phasic responses are referred to as skin conductance responses (SCRs). Both event-related indicators and SCL level were computed as the GSR features (Table 2), including the number of SCRs (nSCR), latency of the first SCR (Latency), sum of SCR amplitudes (AmpSum), average phasic driver (SCR), area of phasic driver (ISCR), maximum value of phasic activity (PhasicMax), and mean tonic activity (Tonic). Continuous decomposition analysis was applied to decompose the skin conductance data into its tonic and phasic components [66]. The minimum amplitude threshold is defined as 0.01 mus. The task duration after the first stimulus is defined as the response window (length: 5 min).

### 3.3. Statistical Analysis Method

The hypothesis testing for comparing mean values between two related samples is used to examine whether there are statistical differences in subjective reports, task performance, and physiological indicators under the two attentional states. At first, the following two conditions are examined to determine whether the paired sample *t*-test can be used: (1) There is no outlier in the D-value (difference value) of the paired dependent variables. (2) The D-value of the paired dependent variables obeys normal distribution. Otherwise, the non-parametric test method (Wilcoxon’s sign rank test) will be used. Since this sample size is less than 50, the Shapiro–Wilk normality test is used to assess the distribution. If the *p*-value of the Shapiro–Wilk test is less than 0.05, the data is regarded as being not normally distributed.

### 3.4. Prediction Model Development

The following supervised learning algorithms were applied to develop prediction models: Support Vector Machines (SVM), KNearest Neighbor (KNN), Linear Discriminant Analysis (LDA), and Random Forest (RF). Supervised learning was demonstrated to support a quick, accurate, and robust cognitive state detection by learning from individual physiological features [67]. SVM using hyperplanes to separate data points is suitable for classifying psychophysiological data [68]. KNN uses the entire database for prediction based on a similarity measure in the instance space. LDA estimates separating hyperplanes by seeking the direction in feature space where projections of classes have the greatest inner-means distance and the smallest variance. RF classifier applied an ensemble of decision trees for predictions.

**Figure 2:**
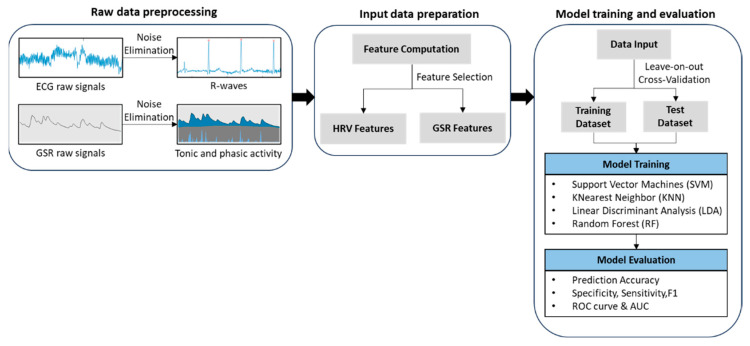
Processes of prediction model development.

Data from a 5-min task under non-fatigued conditions were regarded as a focused state, and data from tasks after physical fatigue was a distracted state. Figure 2 shows the procedures of prediction model development. Feature selection and normalization were performed before model training. The leave-one-out cross-validation (LOO) approach was applied to split the training and test datasets. LOO partitions the data of one subject as the test dataset and the remaining subjects as the training dataset, enabling it to fully use each subject’s sample to reduce the subject bias caused by individual differences [69]. In addition, grid search was used for hyperparameter optimization.

Classification performance was evaluated by overall mean accuracy, sensitivity, specificity, and the area under the receiver operating characteristic (ROC) curve (AUC). The sensitivity is the ratio between the number of positive samples correctly classified as positive to the total number of positive samples. It measures the model’s ability to detect positive samples. The higher the sensitivity, the more positive samples detected. In this study, the distracted samples are defined as positive and concentrated samples as negative. Specificity is the ratio between negative samples and the total number of negative samples. It represents the proportion of the negative samples that were correctly classified. The F1 score, which is the harmonic mean of precision and recall, provides a tradeoff between them. The recall is the ratio between the number of positive samples correctly classified as positive to the total number of positive samples. The precision is the ratio of correctly classified positive samples to the total number of classified positive samples. AUC is a robust measure to compare the performance of classification algorithms. A larger AUC indicates a better classification performance. The proposed classification algorithms and the classification evaluation were implemented in Python 3.7.

## 4. Results

### 4.1. Subjective Reports and Task Performance

**Figure 3:**
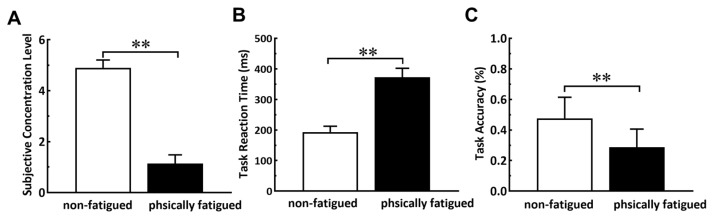
Rating of concentration (**A**), reaction time (**B**), and task accuracy (**C**) before and after physical fatigue. ** *p* < 0.01.

Subjects’ rating of concentration (a five-point Likert scale ranging from 1 (always distracted) to 5 (very concentrated)) after completing the task in the two states is shown in Figure 3A under non-fatigued conditions; subjects reported that they could focus on the task well and their selections were 4 or 5. However, when they completed the task under high physical fatigue, they reported that they were less able to focus on the task, and their options were 1 or 2. The significantly different feedback (*p* < 0.001) of the two conditions showed that fatigue made subjects more likely to be distracted. Specifically, the subjective ratings display abnormal distribution (*p*-value of Shapiro-Wilk test is less than 0.001), and Wilcoxon’s sign rank test is used.

Furthermore, the task performance results also demonstrated that physical fatigue diverted subjects’ attention from tasks. There were significant differences in reaction time (Figure 3B) and accuracy (Figure 3C) between the two states. When the task was completed under physical fatigue, reaction time (*p* < 0.001) increased significantly, and task accuracy (*p* < 0.001) decreased significantly. Specifically, the reaction time demonstrates abnormal distribution (*p*-value of Shapiro-Wilk test is 0.018), with Wilcoxon’s sign rank test being used. The accuracy represents a normal distribution (*p*-value of Shapiro-Wilk test is 0.540), with a paired sample *t*-test being used.

### 4.2. Statistical Analysis

The statistical results of HRV metrics are shown in Table 3. Merely four of the 14 examined metrics were not significant. The four time-domain metrics increased significantly in distraction conditions. Regarding frequency domain metrics, the powers of the low frequency band (LF) and the total power (TP) increased significantly, but there was no significant increase in the powers of high frequency band (HF) and very low frequency band (VLF). The ratio between LF and HF also increased significantly. Neither approximate nor sample entropy of nonlinear metrics significantly differed between the two attentional states.

The statistical results of GSR data are shown in Table 4. One of the examined metrics showed significant differences between concentrated and distracted conditions. When subjects performed the task under physically fatigued conditions, their mean skin electrical activity increased, but there were no significant differences.

### 4.3. Prediction Accuracy and Evaluation of Prediction Models

**Figure 4:**
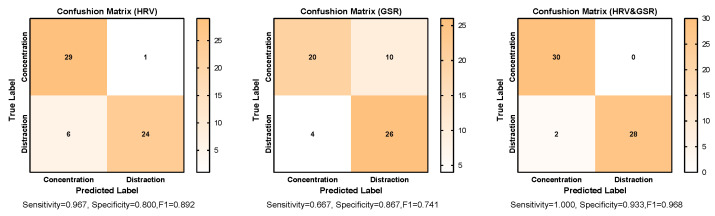
Confusion matrix and evaluation metrics.

**Figure 5:**
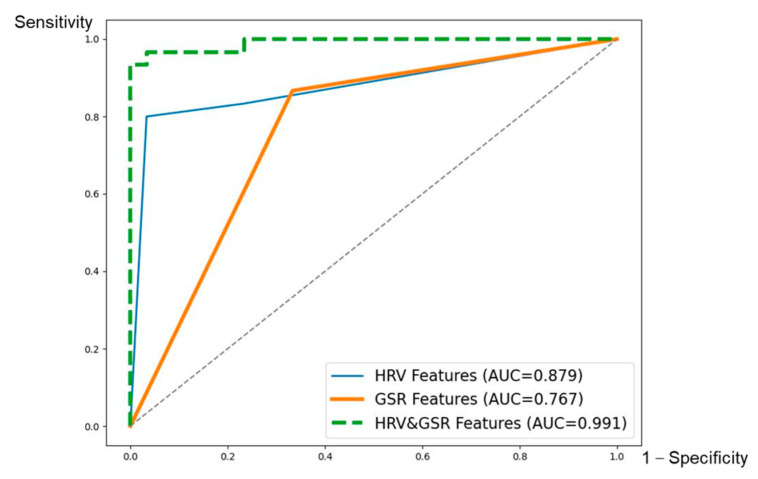
ROC curves of prediction models.

Feature combination was determined based on the statistical analysis results. All features were applied for model training at first; then, features were removed one by one to retrain the model according to the absolute value of their t-values. Features with a smaller absolute t-value would be removed first because they were regarded as contributing less to distinguishing the two conditions. The best prediction accuracy and the corresponding feature combination are shown In Table 5; the confusion matrix and evaluation metrics are shown in Figure 4, and Figure 5 shows the ROC curve. Each column of the confusion matrix represents the instances in a predicted level, while each row represents the instances in a true level. The values of the diagonal elements represent the proportion of correctly predicted levels.

Applying eight HRV features could achieve the best prediction accuracy (88.33%) through the kNearest Neighbor (KNN) algorithm (k = 2). The model has a relatively high sensitivity value (0.967), and its specificity is 0.800, indicating that the model could perform well in detecting distracted subjects. Also, the model has a high AUC value of 0.879.

Applying seven GSR features obtained the highest accuracy (76.67%), also through the KNN algorithm (k = 1). The mean prediction accuracy was less than 80%, and its sensitivity (0.667) is poor, which indicates that the model would fail to identify many distracted samples. The AUC is also poor, with a value of 0.767.

The prediction accuracy was improved when the two kinds of physiological signals were combined. The SVM (RBF kernels, C = 32, γ = 0.5) algorithm achieved the best accuracy of up to 96.67%, with 17 features in total. The model also performed well in specificity (0.993), sensitivity (1.000), and AUC (0.991).

## 5. Discussion

### 5.1. Physiological Parameters and Inattention

Among the HRV time-domain features, the results showed significant differences in mRR, SDRR, RMSSD, and PNN50 between the focused and distracted states. These metrics significantly increased under distraction, which is consistent with the previous finding that HRV decreased when a person maintained sustained attention [38,70]. Also, a previous study reported that mRR is an important feature for distinguishing attention and distraction [35]. Our results show that mRR was statistically significant, but it was not included in the optimal feature combination when the predictive model achieved the highest accuracy. Other time domain features, namely SDRR and PNN50, were shown to be more important in our study. The difference may be due to the different attentional tasks and factors causing distraction between the studies.

Regarding frequency domain features, VLF and HF were insignificant; TP, LF, and the ratio of LF/HF and normalized power were significantly different between the two attentional states. The low and high frequency corresponds to sympathetic and parasympathetic activity, respectively. The sympathetic nervous system is active under stress, fear, and emergency status, while the parasympathetic system will be active under relaxed status [33]. The results showed a significant increase in LF under fatigued conditions, i.e., sympathetic activity was enhanced, which is probably because it is more difficult for subjects to focus on the experimental task when tired. That is, they were experiencing a stressed status. In addition, low frequency has been demonstrated to be a psychophysiological index of effort allocation [71]. It is consistent with our results that LF was significantly increased under distraction and had a large t-value, and LF was also one of the features which is needed to obtain the highest prediction accuracy. The study in [35] reported the importance of VLF, but our results are inconsistent: VLF was not significantly different between concentration and distraction. The inconsistency may also come from the various experimental tasks and distraction causes. Driving distraction studies found that the entropy of ECG signals was sensitive [37,38], but there were no significant differences in the entropy features in this study. This may be because the construction condition is essentially different from the driving task.

The differences in the electrical skin metrics between the two states were less significant than the cardiac metrics. GSR also correlates with the sympathetic nervous system and is related to emotional arousal [40]. It would be more difficult for subjects to focus under fatigue [16]; as a result, subjects may be aroused, and their sympathetic nerves will be enhanced. The electrical skin activity may increase. Moreover, an increased skin conductance response has been demonstrated in the distracted group subjects [46,47]. Our results align with the inference, with an increase in the mean value of the SCR, and a decrease in the mean value of the Tonic. Although most of the GSR metrics were not statistically significant, these features played a key role in predicting distraction. The prediction accuracy was maximized when all GSR features were used. Although GSR features were less predictive than HRV features, combining them with HRV features improved prediction accuracy greatly when combing the two kinds of physiological signals.

### 5.2. Performance of Supervised Learning Algorithms

Previous studies demonstrated that supervised machine learning could be a promising approach for performing cognitive state detection based on physiological features (e.g., [35]). SVM and KNN performed better in our results (Table 5). SVM is suitable for high-dimensional non-Gaussian distribution data, and many psychophysiological data have these characteristics [68]. However, SVM may not perform well in training large-scale datasets [72]. KNN is reported to be compatible with large-scale samples [73]. It could be considered an alternative to SVM when dealing with a considerable sample size. Furthermore, KNN is robust to noisy data, and it is a simple algorithm demanding a low computational cost. These advantages also make it a promising approach for inattention detection when considering its industrial application.

The required number of physiological features needed is relatively small (Table 5), with 8 features needed when using HRV metrics alone, and it could achieve a good accuracy (88.33%); 7 features when using GSR metrics alone, but its accuracy is unsatisfactory (76.67%); and 17 features when using combined features of HRV and GSR, and the accuracy is high (96.67%). The results indicate the advantages of using HRV features alone in construction job sites. Demanded data can be collected by a single sensor (ECG sensor), reducing the data collection difficulty in workplaces compared to using multiple sensors. The small number of needed features also means ECG data can be processed quickly and facilitate low-cost real-time monitoring. Using galvanic skin features alone is not recommended because the accuracy is too low. Two sensors should be applied, and more features must be computed to yield higher accuracy. It might also be feasible because, currently, there are already watches simultaneously monitoring both cardiovascular and galvanic electricity.

## 6. Conclusions, Limitations and Prospects

This study applies electrocardiograph (ECG) and galvanic skin response (GSR) sensors to monitor construction workers’ inattention induced by physical fatigue. The results show that using HRV features alone could obtain a prediction accuracy of 88.33% through the KNN algorithm; using GSR features alone achieved an accuracy of 76.67% through the KNN algorithm; combining HRV and GSR features improved the accuracy to 96.67% through the SVM algorithm. The results indicate that ECG sensors alone or the integration of ECG and GSR sensors can be considered to be applied in job sites to monitor construction workers’ inattention. Future studies are suggested to validate the performance of ECG and GSR sensors in larger samples because the current results were based on a small sample size. In addition, the experiment was conducted in a laboratory rather than a realistic workplace because it would be difficult to control the irrelevant variables in the real construction site. Virtual reality (VR) has the potential to duplicate real-world scenarios; however, this would currently require geometrically modeling each observable element in the scenes, assembling them into a virtual world, and achieving photo-quality rendering, which all requires long development time and high computational costs. Subjects might even suffer VR sickness walking in such a virtual environment. With the development of virtual reality technology, realistic environments could be simulated to conduct research in the future.

## Figures and Tables

**Table 1:** HRV features employed in this work and their descriptions.

HRV Features	Unit	Description
*Time-domain features*		
mRR	[ms]	The mean of RR intervals
SDRR	[ms]	The standard deviation of RR intervals
RMSSD	[ms]	The square root of the mean squared differences between successive RR intervals
pNN50	[%]	Number of interval differences of successive RR intervals greater than 50 ms
*Frequency-domain features*		
VLF	[ms^2^]	Absolute powers of very low frequency band (0–0.04 Hz)
LF	[ms^2^]	Absolute powers of low frequency band (0.04–0.15 Hz)
HF	[ms^2^]	Absolute powers of high frequency band (0.15–0.4 Hz)
TP	[ms^2^]	The total energy of RR intervals
LF/HF	[n.u.]	The ratio between LF and HF band powers
nLF	[n.u.]	Normalized low frequency power
nHF	[n.u.]	Normalized high frequency power
*Nonlinear features*		
SD2/SD1	-	Ratio between SD2 and SD1
ApEn	-	Approximate entropy
SampEn	-	Sample entropy

**Table 2:** GSR features employed in this work and their descriptions.

GSR Features	Unit	Description
SCR	[muS]	Average phasic driver within response window
nSCR	-	Number of significant SCRs within response window
ISCR	[muS∗s]	Area (i.e., time integral) of phasic driver within response window
Latency	[s]	Response latency of first significant SCR within response window
AmpSum	[muS]	Sum of SCR-amplitudes of significant SCRs within response window
PhasicMax	[muS]	Maximum value of phasic activity within response window
Tonic	-	Mean tonic activity within response window

**Table 3:** Statistic analysis of HRV metrics.

HRV Features	Median (P_25_, P_75_) or Mean ± SD		*p*-Value of Shapiro-Wilk Test	t (z)	*p*
	Condition1	Condition2			
mRR	884.06 (869.48, 921.66)	894.45 (873.34, 945.34)	0.004 ^b^	(−2.026)	0.043 *
SDRR	22.86 ± 6.97	25.78 ± 5.29	0.065 ^a^	−3.425	0.002 **
RMSSD	26.85 ± 7.72	28.62 ± 8.19	0.257 ^a^	−2.242	0.033 *
PNN50	6.59 ± 6.10	8.84 ± 8.13	0.741 ^a^	−2.766	0.010 *
VLF	22.39 (12.57, 53.25)	32.56 (27.92, 72.17)	0.002 ^b^	(−1.450)	0.147
LF	105.18 (73.20, 296.07)	291.91 (173.14, 365.52)	0.008 ^b^	(−3.445)	0.001 **
HF	213.87 ± 160.59	224.99 ± 146.28	0.978 ^a^	−1.132	0.267
TP	432.39 ± 276.46	594.09 ± 196.07	0.066 ^a^	−3.941	<0.001 **
LF/HF	1.94 (0.44, 3.42)	0.72 (0.45, 1.79)	0.005 ^b^	(−2.931)	0.003 **
nLF	41.70 (30.64, 64.04)	65.93 (30.47, 77.23)	0.002 ^b^	(−2.499)	0.012 *
nHF	58.27 (35.88, 69.09)	34.02 (22.72, 69.51)	0.002 ^b^	(−2.499)	0.012 *
SD1/SD2	1.45 (1.00, 1.55)	1.41 (1.17, 1.79)	<0.001 ^b^	(−2.170)	0.030 *
ApEn	1.14 (1.10, 1.18)	1.12 (1.10, 1.15)	0.009 ^b^	(−0.504)	0.614
SampEn	1.80 (1.68, 1.87)	1.78 (1.72, 1.89)	<0.001 ^b^	(0.298)	0.766

Note: * *p* < 0.05, ** *p* < 0.01. P_25_—25% quartiles; P_75_—75% quartiles. ^a^ normal distribution and paired sample *t*-test is used (with mean ± SD and t values reported), ^b^ abnormal distribution and Wilcoxon’s sign rank test is used (with median (P_25_, P_75_) and z values reported); condition1—before physical fatigue, Condition2—after physical fatigue.

**Table 4:** Statistic analysis of GSR metrics.

GSR Features	Median (P_25_, P_75_) or Mean ± SD		*p*-Value of Shapiro-Wilk Test	t (z)	*p*
	Condition1	Condition2			
SCR	0.05 (0.02, 0.12)	0.05 (0.13, 0.10)	<0.001 ^b^	(−0.957)	0.339
nSCR	80.50 (32.25, 113.75)	60.50 (24.00, 126.00)	0.014 ^b^	(−0.119)	0.905
ISCR	14.61 (6.07, 35.71)	16.05(3.85, 30.83)	<0.001 ^b^	(−0.957)	0.339
Latency	1.00 (0.68, 1.75)	3.95 (0.80, 1.35)	<0.001 ^b^	(−3.261)	0.001 **
Tonic	1.22 ± 0.78	1.17 ± 0.85	0.400 ^a^	0.668	0.509
AmpSum	3.41 (0.85, 9.20)	3.33 (0.49, 7.34)	<0.001 ^b^	(−0.915)	0.360
PhasicMax	0.92 (0.67, 2.42)	1.23 (0.53, 2.32)	<0.001 ^b^	(−0.977)	0.329

Note: ** *p* < 0.01. P_25_—25% quartiles; P_75_—75% quartiles. ^a^ normal distribution and paired sample *t*-test is used (with mean ± SD and t values reported), ^b^ abnormal distribution and Wilcoxon’s sign rank test is used (with median (P_25_, P_75_) and z values reported); Condition1—before physical fatigue, Condition2—after physical fatigue.

**Table 5:** Summary of evaluations of prediction models with the best accuracy.

Feature Combination		KNN	SVM	LDA	RF
HRV features *n* = 8	SDRR, PNN50, LF, TP, LF/HF, nLF, nHF, SD1/SD2	88.33% (k = 2)	86.67%	63.33%	80.00%
GSR features *n* = 7	SCR, nSCR, ISCR, Latency, Tonic, AmpSum, PhasicMax	76.67% (k = 1)	58.33%	46.67%	63.33%
HRV and GSR features *n* = 17	mRR, RMSSD, SDRR, PNN50, LF, TP, LF/HF, nLF, nHF, SD1/SD2, SCR, nSCR, ISCR, Latency, Tonic, AmpSum, PhasicMax	86.67% (k = 1)	**96.67%**	91.67%	95.00%

Note: underlining indicates the highest accuracy for the feature combination row, and bolding indicates the highest accuracy of all results.

## Data Availability

The data presented in this study are available on request from the corresponding author.
